# Supplementary material for: Susceptibility of Four Abalone Species, Haliotis gigantea, Haliotis discus discus, Haliotis discus hannai and Haliotis diversicolor, to Abalone asfa-like Virus
Source: Viruses. 2021 Nov 20;13(11):2315. doi: 10.3390/v13112315 (PMC8621809; doi:10.3390/v13112315)
Supplement: Supplementary file 1 [file viruses-13-02315-s001.zip › viruses-1420645-supplementary.pdf]

viruses

MDPI

Supplementary Materials

Susceptibility of Four Abalone Species, *Haliotis gigantea*, *Haliotis discus discus*, *Haliotis discus hannai* and *Haliotis diversicolor*, to Abalone asfa-like Virus

Table S1. Cloning primers.

Primer Name	Forward Primer (5′–3′) *	Primer Name	Reverse Primer (5′–3′) *
MCP-F	gagggatccgaattcATGGCGGCAGGAG-GACCCTTC	MCP-R	ttaagcagagattacTTATGCAGCATATCGCAAGA-TAG
PRV-F	gagggatccgaattcATGTACAGAT-TTACCCAGGAAG	PRV-R	ttaagcagagattacTTAGAGTATGATTGG-GAAGTCC

*: Lower case letters indicate In-Fusion cloning sequence. Upper case letters indicate gene-specific sequence.

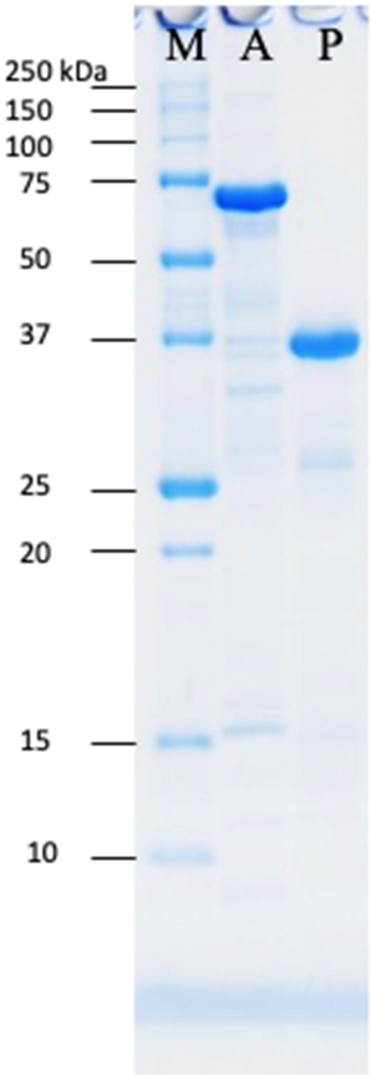

Figure S1. Electrophoretic image of purified His-tagged recombinant protein. M: molecular marker, A: AbALV MCP, P: PRV-2 σ1.
